# The Impact of Insurance and Marital Status on Survival in Patients with Nasopharyngeal Carcinoma

**DOI:** 10.3390/biology9040084

**Published:** 2020-04-22

**Authors:** Chih-Chun Wang, Ching-Chieh Yang, Shyh-An Yeh, Chung-I Huang, Tzer-Zen Hwang, Chuan-Chien Yang, Yu-Chieh Su

**Affiliations:** 1 Department of Otolaryngology, E-Da Hospital, Kaohsiung 824, Taiwan; ccw5969@yahoo.com.tw (C.-C.W.); lord.su@msa.hinet.net (C.-C.Y.); 2 Department of Radiation Oncology, Chi-Mei Medical Center, Tainan 710, Taiwan; cleanclear0905@gmail.com; 3 Department of Pharmacy, Chia-Nan University of Pharmacy and Science, Tainan 717, Taiwan; 4 Department of Radiation Oncology, E-Da Hospital, Kaohsiung 824, Taiwan; yehsa@hotmail.com; 5 School of Medicine, I-Shou University, Kaohsiung 824, Taiwan; hepatomasu@me.com; 6 Department of Radiation Oncology, E-Da Cancer Hospital, Kaohsiung 824, Taiwan; anoldman@gmail.com; 7 Division of Hematology-Oncology, Department of Internal Medicine, E-Da Hospital, Kaohsiung 824, Taiwan

**Keywords:** Surveillance, Epidemiology and End Results, SEER, nasopharyngeal carcinoma, insurance status, marital status

## Abstract

Objective: This study aimed to explore the influence of social support on the survival outcomes of patients with nasopharyngeal carcinoma (NPC). We examined whether the combined proxy influenced whether patients were more likely to receive radiotherapy. Methodology: data were collected from the 18 registries of the National Cancer Institute’s Surveillance, Epidemiology, and End Results database. The association between both insurance status and marital status and disease-specific survival rates were evaluated with a multivariate Cox proportional-hazards regression model to calculate the hazard ratios and associated confidence intervals. Odds ratio (OR) computed by logistic regression was also used to examine the relationship between the receipt of radiotherapy and insurance and marital status. Results: insured and uninsured patients differed significantly in T-stage, N-stage, M-stage, radiotherapy use, race, and marital status. The uninsured-non-married patients showed the lowest 5-year disease-specific survival rates. We further found unmarried patients with either Medicaid (OR, 0.40), or no insurance (OR, 0.24) had lower odds of receiving radiotherapy than those with insurance at diagnosis. Conclusions: uninsured-unmarried NPC patients had a significantly higher risk of distant metastasis at diagnosis, poorer 5-year disease-specific survival, and were less likely to receive radiotherapy than insured-married patients.

## 1. Introduction

A recent study using the National Cancer Institute (NCI) Surveillance, Epidemiology, and End Results (SEER) database found significantly poorer cancer-specific survival outcomes for unmarried patients compared to married patients in an analysis of the ten leading causes of cancer-related deaths, even after adjustment for demographic and clinical factors. Greater social support, such as that provided by marital status, has been suggested as a primary driver for the negative relationship between being married and cancer mortality, particularly because married cancer patients are more likely than unmarried patients to be diagnosed at an earlier disease stage and to receive curative treatment [1,2,3]. One reason why marital status is important is that social support is a vital component of patient cancer screening, aggressive treatment, and follow-up care. However, less well studied is the contribution of economic resources to marriage-associated survival differences, despite the potential for unmarried patients to generally have fewer economic resources, such as income and health insurance, than married patients.

In addition to marital status, a number of studies have reported that uninsured and Medicaid insured patients with breast, colorectal, cervical, lung, or head and neck cancer have either higher mortality or lower survival outcomes than do patients with private insurance, even after adjusting for other factors [4,5,6,7]. From a clinical perspective, patients without insurance have much more difficulty in obtaining adequate treatment for their condition, potentially delaying adjuvant therapy, receiving less than the standard dose of chemotherapy, and increasing their mortality risk. Qiu et al. revealed that the patients with gastric adenocarcinoma have insurance exhibiting better survival rate than those uninsured, and they conjectured that insurance status may impact the survival disparity between married and unmarried patients [8]. As mentioned above, survival from cancer appears to be associated with a complex social support system, of which insurance status and marital status are important parts. While ensuring adequate health insurance for all is an important step, additional measures must be taken to address cancer survival disparities. However, not presently known is the combined effect of both marital status and insurance status on survival in cancer patients, although recent papers have explored each variable separately. In the present study, we combined the insurance and marital status to evaluate the interaction effect in patients with nasopharyngeal carcinoma (NPC), which may help to establish individual therapy strategy and improve the survival.

Nasopharyngeal carcinoma (NPC), a malignant head and neck cancer, is more prevalent in Southeast Asia, North Africa, the Middle East, and Alaska than in Caucasian populations, with an incidence of nearly 1 case per 100,000 person-years [9]. The Chinese population is most affected by NPC, accounting for nearly 43% of NPC patients in the US [10]. More importantly, radiotherapy and chemotherapy are the main treatments for NPC and have satisfactory therapeutic efficacy. Undergoing multiple courses of radiochemotherapy requires intensive heath care and can be expensive. Therefore, it is vital to understand the role of social support in the treatment and survival of NPC patients. However, to date, few papers have examined this issue in detail. To our knowledge, this is the first study of US patients diagnosed with NPC using the SEER database for 2007–2013 to determine the influence of both marital status and insurance status on 5-year survival and receipt of radiotherapy.

## 2. Materials and Methods

### 2.1. Data and Patient Selection

We identified patients with NPC (the 3rd edition of the International Classification of Diseases for Oncology [ICD-O-3] codes) diagnosed in the 18 registries of the SEER database, which is sponsored by the NCI and covers 34.6% of the US population. The SEER database provides accurate, timely, and continuous data on patient demographics, clinicopathologic details, and cancer survival. The patients were pathologically diagnosed with primary NPC between January 1, 2007, and December 31, 2013, based on the SEER*Stat software, version 8.3.4. Excluded were 1300 patients who did not meet the study criteria, being one of the following: age at diagnosis ≤18 years or unknown; insufficient or unknown stage, histology and treatment; or lacking clear records on marital status and insurance status.

### 2.2. Study Variables

The following variables were analyzed: age, gender, T-stage (T1, T2, T3, or T4), N-stage (N0, N1, N2, or N3), M-stage (M0 or M1), radiotherapy (yes or no), WHO category (WHO I–II or WHO III), and race (non-black or black). Marital status was classified as either married or unmarried, the latter combining those who were single (never married), divorced (divorced or separated), or widowed; insurance status was defined as insured, on Medicaid, or uninsured. Using this information, the combined factor of marital status and insurance status was defined into six groups: insured-married, insured-unmarried, Medicaid-married, Medicaid-unmarried, uninsured-married, and uninsured-unmarried. The variables of insurance status and marital status were determined at diagnosis. The primary survival outcome of this study was disease-specific survival, defined as the period from initial diagnosis to date of death due to NPC or last date known alive. A secondary outcome was receipt of radiotherapy.

### 2.3. Statistical Analysis

Patient demographic characteristics, clinicopathologic features, radiotherapy use, and marital status were compared according to insurance status. Continuous variables were reported as means and standard deviations with one-way ANOVA, and categorical variables as frequencies and percentages, examined by Pearson’s chi-square test or Fisher’s exact test. The cumulative 5-year disease-specific survival rate was plotted using Kaplan-Meier curves and the survival differences were compared using the log-rank test. We computed hazard ratios (HRs) and their 95% confidence intervals (CIs) applying univariate and multivariable Cox proportion hazards regression models to evaluate the influence of the combination of insurance status and marital status on the 5-year disease-specific survival rates; the multivariable analysis was adjusted for all baseline characteristics. Moreover, we used multivariate logistic regression to quantitatively confirm the association between the combination of insurance status and marital status and the receipt of radiotherapy. The estimated odds ratios (ORs) were reported and adjusted for all baseline variables as well. All statistical analyses were conducted using IBM SPSS Statistics for Windows, version 20 (IBM Corp., Armonk, NY, USA). A *p*-value less than 0.05 was considered statistically significant.

## 3. Results

**Figure 1:**
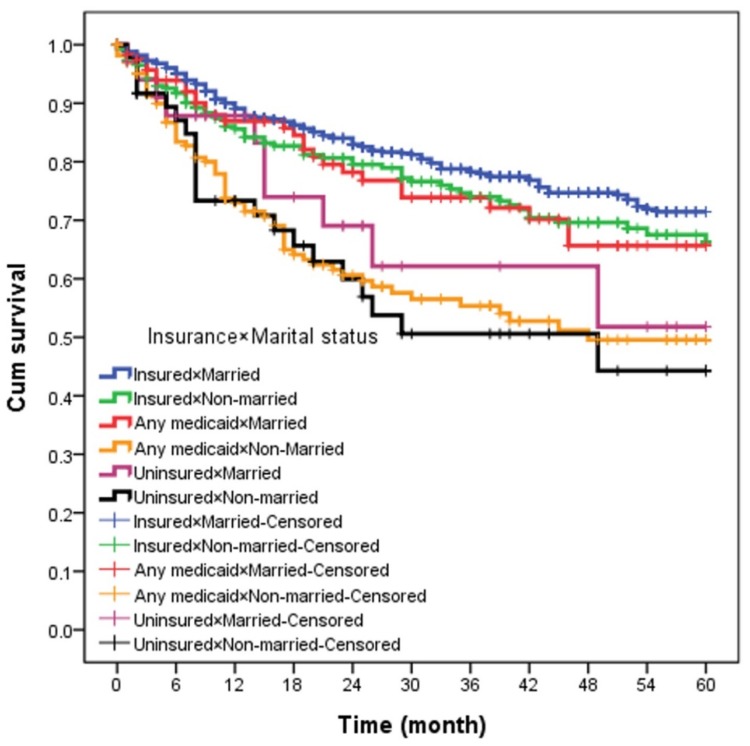
The 5-year overall survival according to insurance × marital status.

The demographic and clinical data for 1300 NPC patients in the study population are summarized in Table 1. Most, or 825 (63.5%) patients, were married, and 475 (36.5%) were unmarried; 933 (71.8%) had private insurance, 285 (21.9%) had Medicaid and 82 (6.3%) had no insurance. Among these patients, the insured patients were the oldest (mean age 54 years, *p* < 0.001). Higher proportions of those were male, and particularly up to 80.5% of the uninsured were male. The highest proportion of early T-stage were among the insured patients compared to those on Medicaid or uninsured (38.4% vs. 34.4–26.8%, *p* < 0.001), as were early N-stage and M-stage (26.6% vs. 20.7–17.9%, *p* < 0.001; 90.1% vs. 83.9–72.0%, *p* < 0.001 respectively). Correspondingly, the uninsured were more frequently represented by later T-stage, N-stage, and M-stage. The rate of receiving radiotherapy was 90.1% in insured patients and 75.6% in uninsured ones (*p* < 0.001). A higher proportion of uninsured patients were in the WHO I-II category (64.6% vs. 51.9–58%) while insured patients were more likely to be non-black (*p* < 0.001). A higher proportion of unmarried patients were uninsured (58.5% vs. married/insured%). Insured-married NPC patients had a superior 5-year disease-specific survival compared with other groups (log-rank test, *p* < 0.001). The 5-year disease-specific survival was 75%, 71%, 68%, 53%, 50%, and 45% for the insured-married, insured-unmarried, Medicaid-married, Medicaid-unmarried, uninsured-married, and uninsured-unmarried, respectively (Figure 1).

Table 2 shows the univariate analysis of the effect of the combination of insurance status and marital status. Unmarried patients with either Medicaid or no insurance had significantly poorer 5-year disease-specific survival than the other four groups. The hazard ratios were 2.42 (95% CI, 1.80 to 3.25, *p* < 0.001) and 2.66 (95% CI, 1.68 to 4.21, *p* < 0.001) for Medicaid-unmarried and uninsured-unmarried patients, respectively, compared to insured-married patients. In a multivariate logistic regression model adjusted for age, gender, T-stage, N-stage, M-stage, WHO category and race, unmarried patients were less likely to receive radiotherapy if they had either Medicaid (OR, 0.40, 95% CI, 0.24 to 0.69, *p* = 0.001) or no insurance (OR, 0.24, 95% CI, 0.11 to 0.53, *p* < 0.001) in comparison with those insured at diagnosis. Those who were late-T stage vs. early-T stage (OR, 2.81, 95% CI, 1.30 to 6.07, *p* = 0.009), who were WHO III category vs. WHO I-II (OR, 1.72, 95% CI, 1.15 to 2.56, *p* = 0.007) and who were non-black vs. black were also more likely to receive radiotherapy (Table 3).

In terms of multivariate analysis with Cox hazards regression analysis, when controlling for the variables validated as independent prognostic factors, the combined variable of insurance status and marital status was still an independent predictor of 5-year disease-specific survival. Uninsured-unmarried patients had the highest and significantly increased risk of NPC-related death relative to insured-married patients (HR, 2.82, 95% CI, 1.74 to 4.58, *p* < 0.001). Those with Medicaid-unmarried (HR, 2.76, 95% CI, 2.03 to 3.76, *p* < 0.001) and those insured-unmarried (HR, 1.52, 95% CI, 1.12 to 2.06, *p* = 0.007) also had worse 5-year survival than the insured-married. Interestingly, insurance status provided some protection for unmarried patients, decreasing HR value from 2.82 to 1.52. The other predictors independently associated with lower survival were increased age, male gender, advanced T-stage, N-stage, and M-stage, no receipt of radiotherapy, WHO I–II category, and black race, as shown in Table 4.

## 4. Discussion

To our best knowledge, this study is the first to investigate the combined influence of insurance status and marital status on 5-year survival in patients diagnosed with NPC between 2007 and 2013 years using a population-based cohort. We also analyzed the influence of these factors on the likelihood of receiving radiotherapy. Our results showed that the 5-year disease-specific survival was 75% in the insured-married group and 45% in the uninsured-unmarried group. In multivariate analysis, after adjustment for age, gender, T-stage, N-stage, M-stage, WHO category, and race, the combination of insurance status and marital status remained an independent prognostic factor. Those uninsured-unmarried had the worst survival outcome and were least likely to obtain radiotherapy.

Our results are consistent with the research of Wu et al. who had found that unmarried NPC patients were more likely than married ones to have worse prognosis and to die sooner [11]. Others have found that marriage is related to diagnosis at early-T and early-N stage, with a higher proportion of distant disease in unmarried NPC patients [12]. Our results are also similar to those of previous studies of other malignancies, such as Osborne et al., who found that unmarried women with breast cancer were more likely to be diagnosed at later stages and were less willing than married women to receive definitive therapy; Ayal et al. found a similar marital effect in other cancers [13,14]. More specifically, Xu et al. found a survival benefit in non-Hispanic white and Chinese American patients, with unmarried groups having significantly poorer CSS/OS [12]. A primary explanation for the relationship between marital status and improved cancer survival outcomes is the social/family support and monitoring from the spouse [15,16]. Spouses may encourage their partners to obtain more aggressive treatment, provide emotional aid, and enhance caregiving. Furthermore, a previous study found that the intervention effect of sociomedicine improved the survival rate in early and middle stages of some malignant tumors, including intestinal cancer, mammary cancer, and pulmonary cancer [17]. In addition, a previous study revealed that the social support is beneficial for patients who are not proactive about seeking medical care on their health problems [18]. In Asian American populations, many patients with breast or ovarian cancer are reluctant to discuss emotional problems and personal feelings about the disease, so they are not open to seeking help outside of their family [19]. Therefore, it is worth it to investigate the intervention effect of sociomedicine and social support when combined with marriage and insurance on the survival rate in patients with nasopharyngeal cancer.

On the other hand, insurance status has recently shown to be a particularly important part of social support. For example, Hsu et al. showed that insurance status at diagnosis is associated with breast cancer mortality and Walker et al. found that uninsured patients with the ten most deadly cancers were more likely to have worse survival outcomes [4,20]. One of the main explanations is that insured patients can afford the expense of complying with medical recommendations. In contrast, however, even when the above-mentioned proxies for social support are explored individually or taken into the Cox-hazard model as control variables, issues remain. Hence, the contribution of our study is that we examined the relationship between social support, as the combined factor of insurance status and marital status, and survival disparities. We believe that the combined factor of both insurance status and marital status may be a more effective proxy variable for social support than zip-code income, Medicaid use, or other currently used variables. Using insurance status and marital status, we were able to observe the negative effect of being uninsured-unmarried on survival outcomes in NPC patients. Furthermore, in this paper, we also provide empirical issue that uninsured-unmarried patients were less likely to receive radiotherapy, although many prior studies have hinted at this relationship. This point is of particular importance for NPC patients, because costly radiotherapy is a main treatment for NPC.

## 5. Study Limitations

This study had some limitations. First, this study used the SEER database, which provided information on insurance and marital status only at the time of diagnosis or initial treatment. The status of either could change during treatment and follow up. We also did not know the economic status or quality of marriage from the database. Although the patients were married, they could still live apart, and unmarried patients could still have a partner or other living companion. Second, the SEER database does not provide the details of treatment, including chemotherapy regimen and radiotherapy technique. These important factors may affect outcomes in patients. Third, we did not take comorbidities into account, which could also affect the intensity of treatment and consequently treatment outcomes. Finally, owning to the small sample size, although we realize that insurance eligibility policies are not uniform across states, we did not account for these varying policies. We also did not stratify the unmarried into groups of widowed, never married, and divorced. Further studies are needed to elucidate the association between survival and different types of unmarried status and insurance status.

## 6. Conclusions

To sum up, as an important type of social support, having both insurance and a spouse results in a significantly better 5-year disease-specific survival rate for patients diagnosed with NPC in our study. Our results suggest that being both uninsured and unmarried not only increases the risk of NPC mortality, when compared with others, but also represent a newly identified high-risk subgroup with poorer prognosis. The targeted incorporation of social support into the integrated therapy and personalized care of patients with NPC should be a national healthcare priority for both providers and policymakers.

## Figures and Tables

**Table 1:** Baseline characteristics of nasopharyngeal cancer patients according to insurance status, *n* = 1300.

Characteristics	Insured	Any Medicaid	Uninsured	
Variables	*n* (%)	*n* (%)	*n* (%)	*p* Value
Age, years (Mean ± SD)	54 ± 13	49 ± 16	48 ± 10	<0.001
Gender				0.115
Male	649 (69.6%)	200 (70.2%)	66 (80.5%)	
Female	284 (30.4%)	85 (29.8%)	16 (19.5%)	
AJCC cT				<0.001
T1	358 (38.4%)	98 (34.4%)	22 (26.8%)	
T2	302 (32.4%)	67 (23.5%)	25 (30.5%)	
T3	223 (23.9%)	90 (31.6%)	21 (25.6%)	
T4	50 (5.4%)	30 (10.5%)	14 (17.1%)	
AJCC cN				<0.001
N0	248 (26.6%)	51 (17.9%)	17 (20.7%)	
N1	337 (36.1%)	99 (34.7%)	23 (28.0%)	
N2	251 (26.9%)	73 (25.6%)	22 (26.8%)	
N3	97 (10.4%)	62 (21.8%)	20 (24.4%)	
AJCC cM				<0.001
M0	841 (90.1%)	239 (83.9%)	59 (72.0%)	
M1	92 (9.9%)	46 (16.1%)	23 (28.0%)	
Radiotherapy				<0.001
Yes	841 (90.1%)	244 (85.6%)	62 (75.6%)	
No	92 (9.9%)	41 (14.4%)	20 (24.4%)	
WHO category				0.070
WHO I-II	541 (58.0%)	148 (51.9%)	53 (64.6%)	
WHO III	392 (42.0%)	137 (48.1%)	29 (35.4%)	
Race				<0.001
Non black	865 (92.7%)	240 (84.2%)	62 (75.6%)	
Black	68 (7.3%)	45 (15.8%)	20 (24.4%)	
Marital status				<0.001
Married	670 (71.8%)	121 (42.5%)	34 (41.5%)	
Others	263 (28.2%)	164 (57.5%)	48 (58.5%)	

Abbreviation: AJCC, American Joint Committee on Cancer; cT, clinical T stage; cN, clinical N stage, cM, clinical M stage.

**Table 2:** Univariate analysis for 5-year overall survival.

Variables	Total	Event (%)	HR (95%CI)	*p* Value
Insurance × marital status				
Insured × married	670	138 (20.6%)	1	
Insured × non-married	263	65 (24.7%)	1.26 (0.94–1.69)	0.125
Any Medicaid × married	121	28 (23.1%)	1.28 (0.86–1.93)	0.228
Any Medicaid × non-married	164	65 (39.6%)	2.42 (1.80–3.25)	<0.0001
Uninsured × married	34	10 (29.4%)	1.88 (0.99–3.57)	0.054
Uninsured × non-married	48	21 (43.8%)	2.66 (1.68–4.21)	<0.0001

Abbreviation: HR, hazard ratio; CI, confidence interval.

**Table 3:** Adjusted odds ratios for the association between variables and the opportunity of radiotherapy.

Variables	OR (95%CI)	*p* Value
Insurance × marital status		
Insured × married	1	
Insured × non-married	0.77 (0.47–1.26)	0.304
Any Medicaid × married	1.02 (0.50–2.08)	0.937
Any Medicaid × non-married	0.40 (0.24–0.69)	0.001
Uninsured × married	0.62 (0.24–1.60)	0.325
Uninsured × non-married	0.24 (0.11–0.53)	<0.001
Age	0.98 (0.96–0.99)	0.005
Gender		
Male	1	
Female	0.86 (0.57–1.30)	0.491
AJCC cT		
T1	1	
T2	1.19 (0.75–1.88)	0.444
T3	1.00 (0.64–1.57)	0.979
T4	2.81 (1.30–6.07)	0.009
AJCC cN		
N0	1	
N1	1.77 (1.13–2.79)	0.012
N2	2.58 (1.51–4.39)	<0.001
N3	1.60 (0.88–2.89)	0.120
AJCC cM		
M0	1	
M1	0.17 (0.10–0.27)	<0.001
WHO category		
WHO I–II	1	
WHO III	1.72 (1.15–2.56)	0.007
Race		
Non black	1	
Black	0.59 (0.35–0.99)	0.046

Abbreviation: AJCC, American Joint Committee on Cancer; cT, clinical T stage; cN, clinical N stage, cM, clinical M stage; OR, odds ratio; CI, confidence interval.

**Table 4:** Multivariate analysis for 5-year mortality.

Variables	HR (95% CI)	*p* Value
Insurance × marital status		
Insured × married	1	
Insured × non-married	1.52 (1.12–2.06)	0.007
Any Medicaid × married	1.41 (0.93–2.13)	0.105
Any Medicaid × non-married	2.76 (2.03–3.76)	<0.001
Uninsured × married	1.27 (0.65–2.46)	0.477
Uninsured × non-married	2.82 (1.74–4.58)	<0.001
Age	1.05 (1.04–1.06)	<0.001
Gender		
Male	1	
Female	0.69 (0.52–0.90)	0.007
AJCC cT		
T1	1	
T2	1.10 (0.82–1.48)	0.509
T3	1.61 (1.21–2.14)	0.001
T4	1.88 (1.27–2.78)	0.001
AJCC cN		
N0	1	
N1	1.30 (0.96–1.77)	0.090
N2	1.61 (1.16–2.25)	0.004
N3	2.01 (1.39–2.89)	<0.001
AJCC cM		
M0	1	
M1	2.98 (2.25–3.93)	<0.001
Radiotherapy		
Yes	1	
No	2.31 (1.76–3.04)	<0.001
WHO category		
WHO I–II	1	
WHO III	0.75 (0.59–0.96)	0.023
Race		
Non black	1	
Black	1.45 (1.05–2.00)	0.022

Abbreviation: AJCC, American Joint Committee on Cancer; cT, clinical T stage; cN, clinical N stage, cM, clinical M stage; HR, hazard ratio; CI, confidence interval.
